# Comprehensive landscapes of the causal network between immunity and sarcopenia

**DOI:** 10.3389/fimmu.2024.1443885

**Published:** 2024-08-20

**Authors:** Mingchong Liu, Jiaming Wang, Yi Han, Xiao Fu, Yutao Pan, Chensong Yang, Guixin Sun

**Affiliations:** Department of Traumatic Surgery, Shanghai East Hospital, School of Medicine, Tongji University, Shanghai, China

**Keywords:** sarcopenia, immune cell phenotypes, immune factors, Mendelian randomization, network analysis

## Abstract

**Background:**

Inflammaging, an immune status characterized by a sustained increase in pro-inflammatory markers and a decline in anti-inflammatory mechanisms, is a critical risk factor in the development of sarcopenia. Landscapes of the causal relationships between immunity and sarcopenia are needed to understand the mechanism of sarcopenia and provide novel treatments comprehensively.

**Methods:**

We used Mendelian Randomization (MR) as the basic method in this study. By setting immune proteins, immune cells, and sarcopenia as exposures and outcomes alternatively, and then combining them in different directions, we potentially estimated their causal relationships and directions and subsequently mapped the comprehensive causal landscape based on this information efficiently. To further understand the network, we developed a method based on rank-sums to integrate multiple algorithms and identify the key immune cells and proteins.

**Results:**

More than 1,000 causal relationships were identified between immune cell phenotypes, proteins, and sarcopenia traits (p < 0.05), and the causal maps of these linkages were established. In the threshold of FDR < 0.05, hundreds of causal linkages were still significant. The final comprehensive map included 13 immune cell phenotypes and 8 immune proteins. The star factors in the final map included EM CD8br %CD8br, EM DN (CD4- CD8-) %DN, SIRT2, and so on.

**Conclusion:**

By reading the landscapes in this study, we may not only find the factors and the pathways that have been reported and proven but also identify multiple novel immunity cell phenotypes and proteins with enriched upstream and downstream pathways.

## Introduction

Sarcopenia, characterized by the progressive loss of muscle mass, quality, and function with advancing age, is considered an age-related disease with significant implications for overall health and mobility (1). While the specific etiology of sarcopenia remains incompletely understood, aging has inevitably emerged as a significant risk factor in exploring the onset and progression of sarcopenia (2).

Various factors associated with the aging process, such as chronic inflammation, oxidative stress, metabolic disturbances, and hormonal imbalances, may directly or indirectly contribute to the development of sarcopenia (3). In recent years, inflammaging, the chronic, low-grade inflammatory status in older adults, has been attracting increasing attention in geriatrics and gerontology (4). Inflammaging is characterized by a sustained increase in pro-inflammatory markers and a decline in anti-inflammatory mechanisms, leading to a status of chronic and systemic inflammation (5). Inflammaging is thought to relate to the development and progression of various age-related diseases, such as cardiovascular disease, neurodegenerative disorders, and sarcopenia (6).

Recent research has highlighted the intricate interplay between sarcopenia and inflammaging, suggesting that the chronic inflammatory microenvironment may contribute to the development and progression of sarcopenia by disrupting the delicate balance between muscle protein synthesis and breakdown, leading to muscle wasting and impaired muscle function (7). Moreover, the increased pro-inflammatory cytokines, altered immune-cell phenotypes, and activated inflammatory signaling pathways may directly impact muscle tissue, promoting muscle catabolism and inhibiting muscle regeneration processes (8).

Though previous studies suggested the relationships between sarcopenia and inflammaging, how immune factors and cells regulate the development of inflammaging and sarcopenia, and how sarcopenia itself regulates inflammaging and related changes in immune factors and cells, is not fully understood. Many immune factors and cells were identified to contribute to inflammaging and to play roles in sarcopenia (9), but inflammaging as a systematic immunity disorder may be associated with sarcopenia intricately and complexly, and knowledge of several inflammatory proteins and cells may not comprehensively reveal the changes of immunity in patients with sarcopenia. A novel and high-throughput method were required to conveniently and quickly identify the roles of immunity in sarcopenia, which may better reflect the relationships between inflammaging and sarcopenia.

Mendelian randomization (MR) may be a possible method to conveniently and quickly estimate the causality between immunity and sarcopenia (10). In this study, we aim to comprehensively map the causal relationships between immunity, and sarcopenia traits by using MR. By setting immune factors, immune cells, and sarcopenia as exposures and outcomes alternatively, and then combining them in different directions, we can potentially predict their causal relationships and directions, and subsequently map the comprehensive causal landscape based on this information.

## Methods

### Basic principles

This study was an MR study and was reported according to the STROBE-MR (11). Based on the interpretation of MR results, the exposure is estimated to be a potential cause of the outcome, so we further hold the principles that by setting different exposures and outcomes with different traits, we may identify the causal roles and directionalities of these traits and then comprehensively establish the causal map of immunity and sarcopenia.

### Ethics statement

Given the characteristics of GWAS summary data and pQTL data, no personally identifiable information of individual participants was retrieved at any point during or after data collection. There was no necessity for additional ethics approval or consent to participate. Details regarding ethics approval and consent to participate in each study were available in the original studies

### Overall design

In this study, the traits included 731 immune cell phenotypes, 91 immune proteins, and 6 sarcopenia traits. The sarcopenia traits were selected based on the previous suggestion. A total of 142,906 times of MR analyses were performed and 1,000,342 MR models were established. To better describe these analyses, we grouped these analyses as below:

Analysis group A_forward_: exposures-731 immune cell phenotypes; outcomes-6 sarcopenia traits; 4386 times of analyses;

Analysis group A_reverse_: exposures-6 sarcopenia traits; outcomes-731 immune cell phenotypes; 4386 times of analyses;

Analysis group B_forward_: exposures-91 immune proteins; outcomes-6 sarcopenia traits; 546 times of analyses;

Analysis group B_reverse_: exposures-6 sarcopenia traits; outcomes-91 immune proteins; 546 times of analyses;

Analysis group C: 731 immune cell phenotypes and 91 immune proteins were set as exposures and outcomes alternatively; 133,042 times of analyses.

### Datasets and population of immunity

731 immune cell phenotypes and 91 immune proteins were included as immunity traits. The datasets for 731 immune cell phenotypes were from a group of Genome-Wide Association Studies (GWASs) performed on a cohort of 3757 Sardinians (12). This cohort is a part of SardiNIA project, a longitudinal study with 6602 general participants ranging from 18 to 102 years, 57% were females and 43% were males. The SardiNIA project was conducted in the Lanusei Valley in the Ogliastra region of the Sardinian province of Nuoro in 2001. The peripheral blood samples were collected from the participants and processed for flow cytometers (BD FACSCanto II). The cell phenotypes included 188 absolute cell counts, 389 median fluorescence intensities (MFIs) of surface antigens, 192 relative counts, and 32 morphological parameters. All analyses were adjusted for sex, age, and age^2^. The detailed definition and information on each cell phenotype were summarized in **Supplementary Table 1**.

The datasets for 91 immune proteins were from a genome-wide protein Quantitative Trait Loci (pQTL) of 11 cohorts including 14824 individuals (13). All individuals included in these 11 cohorts were of European descent from England, Sweden, Germany, Scotland, Croatia, Estonia, and so on. The periods of recruitment of these studies ranged from 2000 to 2016. All the proteins were measured by using the Olink Target Inflammation panel. The proteomic data of these 91 immune factors were the main traits of this study. All the analyses on these 11 raw cohorts were adjusted for age, sex, and other specific co-factors. The detailed information on 91 immune proteins is shown in **Supplementary Table 2**. The detailed description of datasets and cohorts can be found in **Supplementary Table 3**.

### Datasets and population of sarcopenia traits

6 sarcopenia traits were selected in our study, including hand grip strength (European Working Group on Sarcopenia in Older People [EWGSOP]), hand grip strength (Foundation for the National Institutes of Health [FNIH]), adjusted appendicular lean mass (AALM), moderate-to-vigorous physical activity (MVPA) levels, usual walking pace, and able to walk or cycle unaided for 10 minutes (AWCU10). These traits meet the three main parts of the diagnosis of sarcopenia (14).

The datasets for handgrip strength were collected from a meta-GWASs including 22 cohorts with 254894 participants aged 60 years or older with European descent (15). The earliest study in these 22 cohorts was initiated in 1948, and the latest may be completed in 2020. The cutoff values of EWGSOP and FNIH were used to identify the poor handgrip strength, respectively. 48596 and 20335 individuals were defined as weak muscle strength based on EWGSOP and FNIH criteria, respectively. All participants underwent strict grip strength assessment by hand dynamometers, and the GWAS was adjusted for age and sex.

The datasets of AALM, MVPA, usual walking pace, and AWCU10 were obtained from the summary GWASs data of UK Biobank (16, 17). The detailed description of the definition, value assignment, sample sizes, datasets ID, and cohorts were summarized in **Supplementary Table 3**. Since 2006, the UK Biobank, a vast database, has amassed an unparalleled volume of biological and medical information from 500,000 individuals aged 40 to 69 residing in the UK, as part of an extensive prospective study (https://www.ukbiobank.ac.uk/). The four cohorts for AALM, MVPA, usual walking pace, and AWCU10 were all from the UK Biobank. In the AALM cohort, body composition was assessed using the BIA method, and a total of 450,243 participants aged 48 to 73 were ultimately enrolled. Information on the usual walking pace, MVPA, and AWCU10 was gathered through self-report and wrist-worn accelerometry data. Our analysis included 459,915 individuals for the usual walking pace, 377,234 for MVPA, and 68,537 for AWCU10.

### IV selection

**Figure 1:**
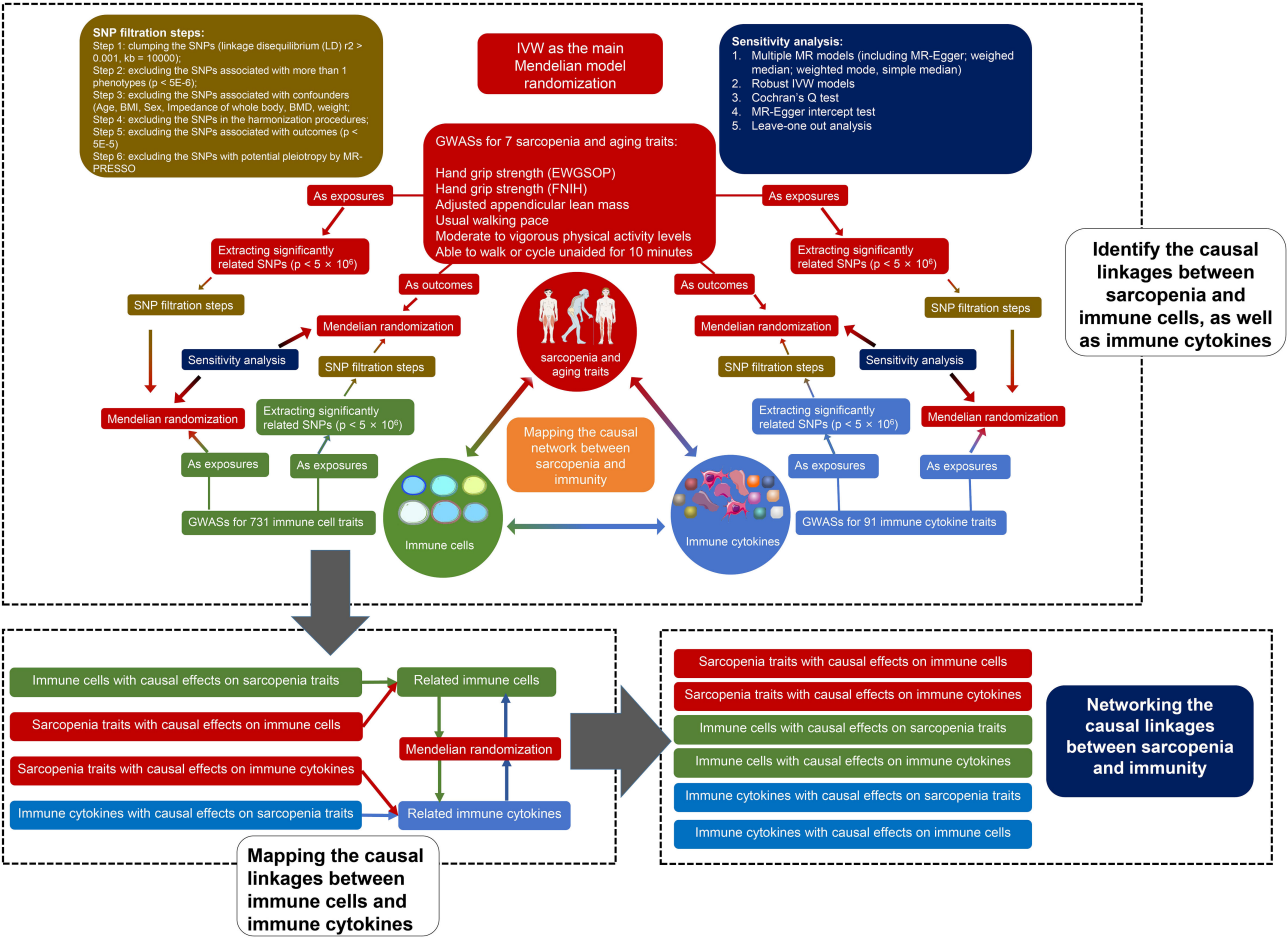
Flow chart of our study.

To satisfy the basic assumptions of the MR and further control the quality of the estimates, six steps of strict instrumental variable (IV) selection were conducted according to the previous protocols (18). The SNP (or cis-SNP) strongly related to GWASs (or pQTL) were selected as IV (p < 5E-6). The detailed IV filtration steps are shown in **Figure 1**. Briefly, after the clumping, the IVs associated with more than 1 trait, associated with confounders, and associated with outcomes were deleted. IVs that were not available in harmonizing and those with potential pleiotropy detected by MR-PRESSO were also excluded.

### MR and sensitivity analyses

7 kinds of MR models were established for each analysis, including random-effects inverse-variance weighted (FE IVW), MR-Egger regression, weighted median (WME), weighted mode (WMD), simple median (SM), fixed effects inverse-variance weighted (FE IVW), and robust inverse-variance weighted (RO IVW). The results of FE IVW were used as the main results and the results of other models were considered as sensitivity analysis. To avoid the bias caused by weak IVs, F statistics and R2 of each IV were calculated. Moreover, the MR-Egger intercept test was performed to assess the directional pleiotropy, and Cochran’s Q test was conducted to detect heterogeneity.

### Network analysis

Considering the immunity and sarcopenia traits as points, and the causality and directionality between them as lines, we may map the causal network between immunity and sarcopenia. Due to the large numbers of immune cell phenotypes and proteins in the network, we developed a novel method based on the rank sum to identify the key traits and quantify their weight. The centrality of each immunity trait, including immune cell phenotypes and proteins, was evaluated firstly using multiple algorithms. The algorithms included Maximal Clique Centrality (MCC), Maximum Neighborhood Component (MNC), Density of Maximum Neighborhood Component (DMNC), Degree, Edge Percolated Component (EPC), Bottleneck, EcCentricity, Closeness, Radiality, Betweenness, Stress, and Clustering Coefficient. Each of these algorithms has its strengths and limitations, and using them individually may have certain restrictions. Therefore, we calculate the ranks of these algorithms through a method of rank sum to comprehensively assess the node centrality.

### Statistics

All p values < 0.05 were considered statistically significant. To avoid the bias caused by multiple tests, the Benjamini-Hochberg (B-H) method was used to adjust the p value and to obtain the adjusted p values (FDR). To map the network of immunity and sarcopenia, all the significant causal linkages were collected. We set two criteria to select the significant linkages by using p values and FDR, respectively. All the analyses were performed using R software version 4.2.2 and R packages “TwoSampleMR”, “MendelianRandomization”, “ComplexHeatmap”, “circlize”, and “RColorBrewer”. The network was mapped by using Cytoscape version 3.10.1.

## Results

### General information

Due to the inadequate numbers of SNPs in immune cell phenotypes, in analysis group A_forward_, 96, 97, 93, 100, 101, and 103 kinds of immune cell phenotypes were excluded for the 6 sarcopenia traits (hand grip strength [EWGSOP], hand grip strength [FNIH], AALM, MVPA, usual walking pace, AWCU10), respectively. Due to the large data size of the results, we summarized the results of each analysis group as result sets. Each result set included the numbers of IVs in each filtration step, the F statistics and R^2^ of each IV, detailed results of each MR model (including beta, odd ratio, 95%CI, p values, and adjusted p values), and detailed results of each sensitivity analyses. The result sets of analysis group A_forward_, analysis group A_reverse_, analysis group B_forward_, and analysis group B_reverse_ were summarized in **Supplementary Tables 4**–**9**, **Supplementary Tables 10**–**15**, **Supplementary Tables 16**–**21**, and **Supplementary Tables 22**–**27**, respectively.

### Causal relationships between immune cells and proteins

To further identify the significant causal linkages between immunity and sarcopenia traits, we select the results of RE IVW models as our main indicators. The relationships with significant p values in RE IVW models in analysis groups A and B were identified and extracted. The immune cell phenotypes and proteins included in these significant causal relationships were collected for further exploring their causality. Similarly, 1910, 3240, 6058, 2673, 2444, and 2832 couples of immune cell phenotypes and proteins were collected according to their causal associations with hand grip strength [EWGSOP], hand grip strength [FNIH], AALM, MVPA, usual walking pace, AWCU10, respectively. The causal relationships between these immune cell phenotypes and proteins were analyzed and the result sets of these analyses (part of analysis C) were summarized in **Supplementary Tables 28**–**33**.

### Causal relationships between immunity and sarcopenia (based on p values)

**Figure 2:**
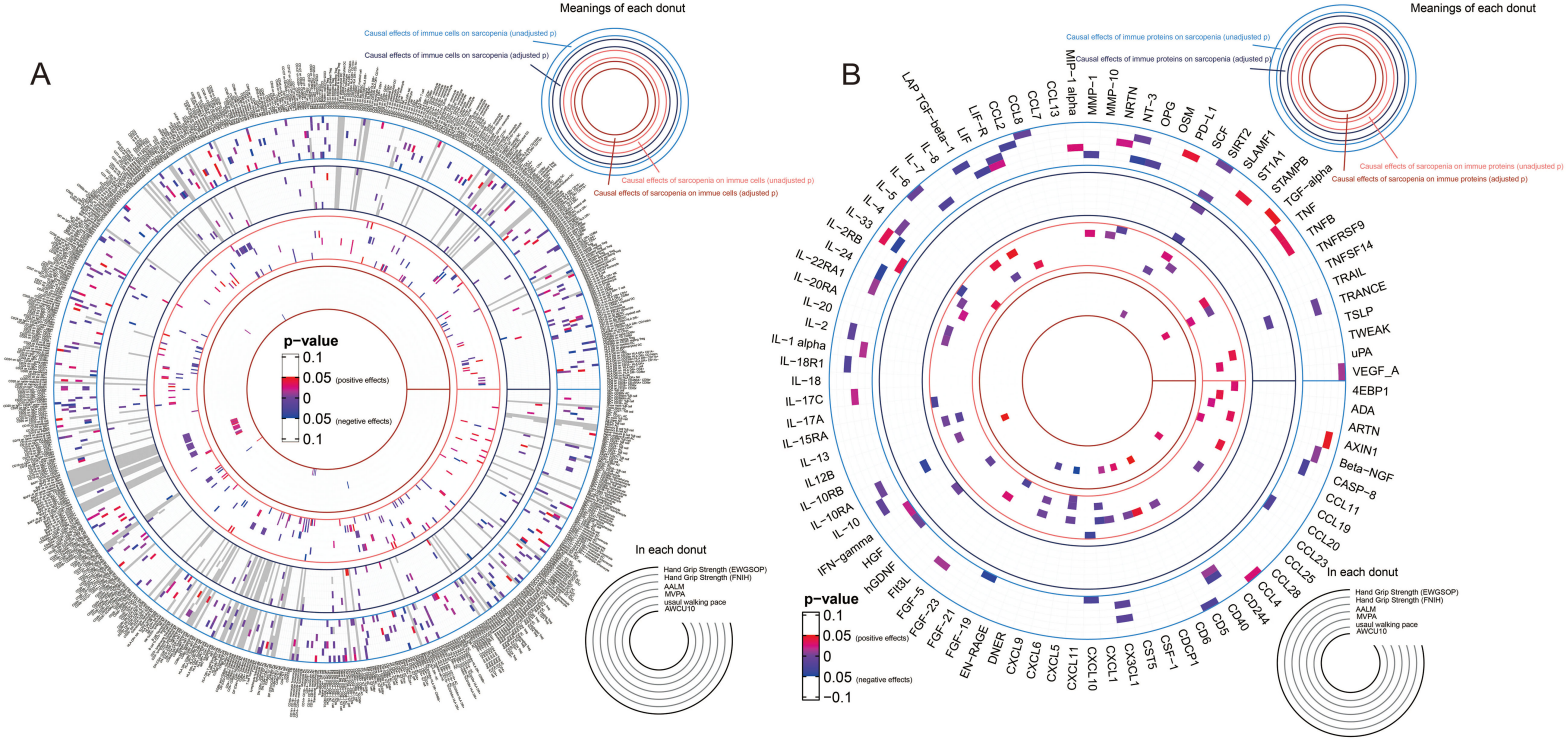
Overall results of the causal relationships between immune cell phenotypes, immune proteins, and sarcopenia traits by using random-effects inverse-variance weighted models. **(A)** the causal relationships between immune cell phenotypes and sarcopenia trait; **(B)** the causal relationships between immune proteins and sarcopenia trait.

Integrating the result sets mentioned above, we extracted all the significant causal relationships and grouped them according to different sarcopenia traits. The overall results of the estimates for the causal relationships between sarcopenia traits and immune cell phenotypes, as well as proteins, were summarized in **Figures 2A** and **B**, respectively.

67 kinds of immune cell phenotypes and 6 kinds of immune proteins were proven to be the potential exposures of hand grip strength (EWGSOP), and reversely, 32 and 4 types of cells and proteins were estimated to be the outcomes of hand grip strength (EWGSOP). Of these cell phenotypes and proteins, 175 significant causal relationships between cell phenotypes and proteins were found (**Supplementary Table 34**). In the relationships related to hand grip strength (FNIH), 58 cell phenotypes and 7 proteins were identified as exposures, and 26 and 13 were suggested as outcomes. In the exploration of the causality between cells and proteins, 278 causal relationships were proven (**Supplementary Table 35**). In the analysis for the relationships between immunity and AALM, 81 cell phenotypes and 12 proteins were estimated as exposures, 44 cell phenotypes and 14 proteins as outcomes, and a total of 485 causal relationships between cells and proteins were proven (**Supplementary Table 36**). As shown in **Supplementary Table 37**, the causal effects of 62 immune cells and 11 immune proteins on MVPA, and the causal effects of MVPA on 44 immune cells and 3 proteins were identified. Between these cell phenotypes and proteins, 291 causal relationships were proven. For the usual walking pace trait, the exposure roles of 51 cell phenotypes and 7 proteins and outcome roles of 37 cell phenotypes and 8 proteins were identified (**Supplementary Table 38**). Lastly, 61 cell phenotypes and 7 proteins were estimated as the exposures of AWCU10, and 66 cell phenotypes and 5 proteins were proven to be the outcomes (**Supplementary Table 39**).

### The causal network between immunity and sarcopenia (based on p values)

**Figure 3:**
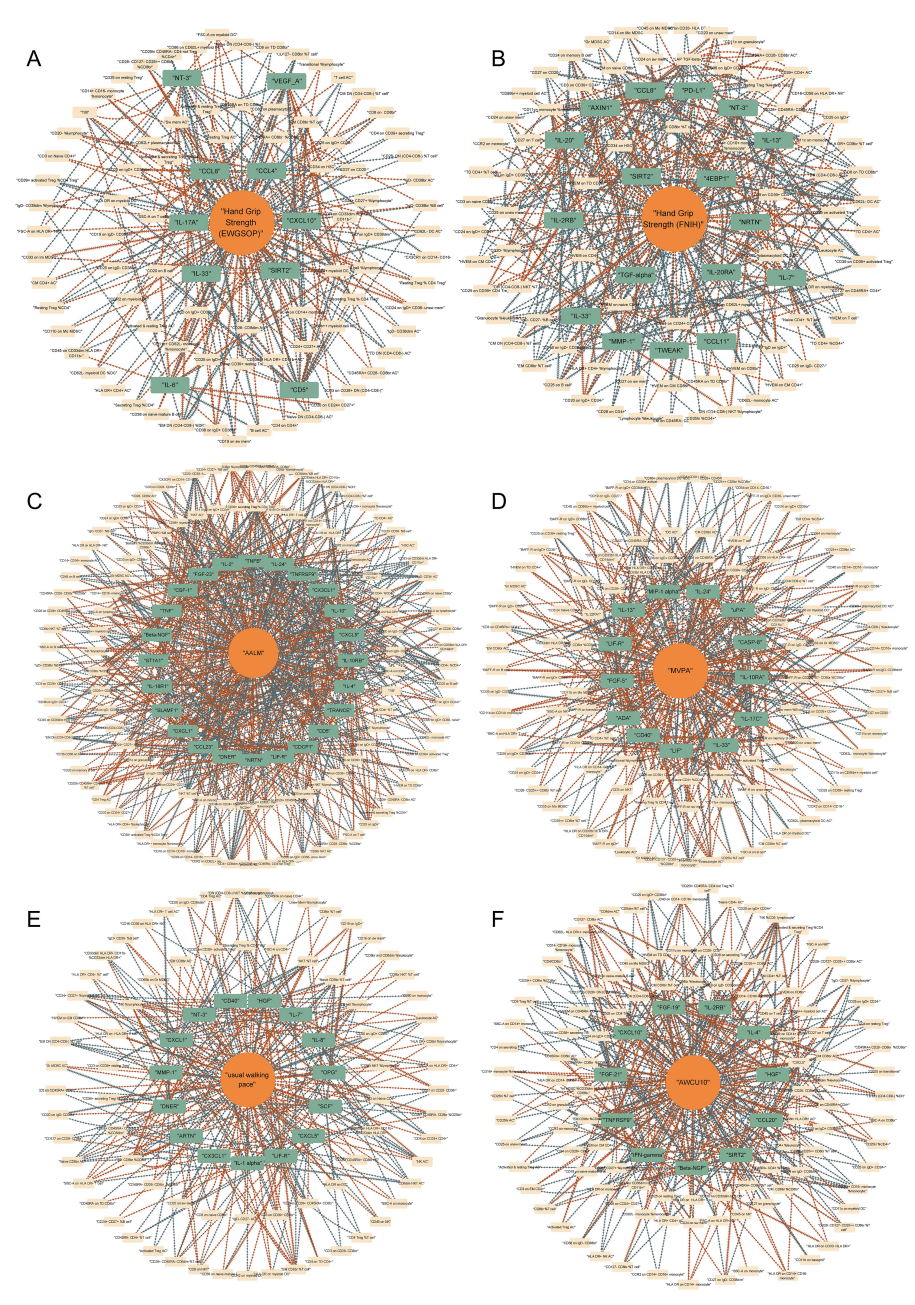
Causal networks between immunity and sarcopenia (based on p values). **(A)** of hand grip strength (EWGSOP); **(B)** of hand grip strength (FNIH); **(C)** of adjusted appendicular lean mass (AALM); **(D)** of moderate-to-vigorous physical activity (MVPA); **(E)** of usual walking pace; **(F)** of able to walk or cycle unaided for 10 minutes (AWCU10).

**Figure 4:**
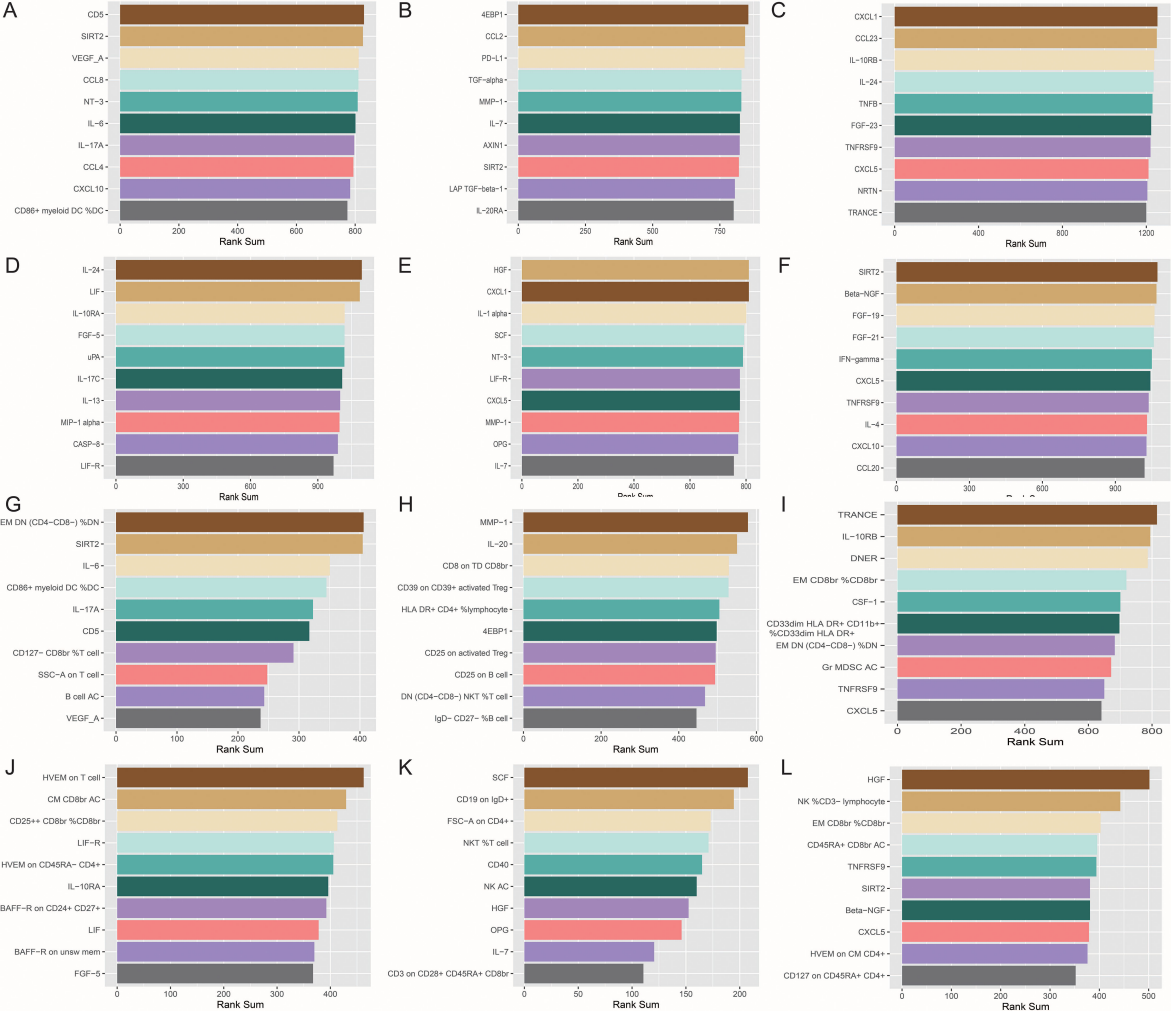
The top 10 immune traits with the highest rank sums in each network. **(A)** in the network (based on p value) of hand grip strength (EWGSOP); **(B)** in the network (based on p value) of hand grip strength (FNIH); **(C)** in the network (based on p value) of adjusted appendicular lean mass (AALM); **(D)** in the network (based on p value) of moderate-to-vigorous physical activity (MVPA); **(E)** in the network (based on p value) of usual walking pace; **(F)** in the network (based on p value) of able to walk or cycle unaided for 10 minutes (AWCU10); **(G)** in the network (based on FDR) of hand grip strength (EWGSOP); **(H)** in the network (based on FDR) of hand grip strength (FNIH); **(I)** in the network (based on FDR) of AALM; **(J)** in the network (based on FDR) of MVPA; **(K)** in the network (based on FDR) of usual walking pace; **(L)** in the network (based on FDR) of AWCU10.

Based on these data, we preliminarily mapped the landscapes of the immunity and sarcopenia traits with a threshold of p values < 0.05. The causal maps for 6 sarcopenia traits were visualized and summarized in **Figures 3A–F**, respectively. Then the network analysis was conducted to further identify the key immunity traits. The results of the centrality of traits evaluated by 12 algorithms in networks for 6 sarcopenia traits were summarized in **Supplementary Tables 40**–**45**. The traits were then ranked according to their weights in each algorithm, and the results were summarized in **Supplementary Tables 46**–**51**. Lastly, the ranks of traits in each algorithm and their rank sums were calculated (**Supplementary Tables 52**–**57**). The top 10 traits with the highest rank sums were visualized in **Figure 4**. The top 3 traits with the highest rank sums in networks of hand grip strength (EWGSOP) were T-cell surface glycoprotein CD5 (CD5), SIR2-like protein 2 (SIRT2), and vascular endothelial growth factor A (VEGF_A). For hand grip strength (FNIH), the top 3 highest rank traits were Eukaryotic translation initiation factor 4E-binding protein 1 (4EBP1), monocyte chemoattractant protein-1 (CCL2), programmed cell death 1 ligand 1 (PD-L1). In the network of AALM, the top 3 traits were C-X-C motif chemokine 1 (CXCL1), C-C motif chemokine 23 (CCL23), and interleukin-10 receptor subunit beta (IL-10RB). For MVPA, interleukin-24 (IL-24), leukemia inhibitory factor (LIF), and interleukin-10 receptor subunit beta (IL-10RA) were the traits with the highest rank sum. In the network of usual walking pace, hepatocyte growth factor (HGF), CXCL1, and interleukin-1-alpha (IL-1 alpha) were the top 3 traits. The traits with the highest rank sums in the network of AWCU10 were SIRT2, beta-nerve growth factor (Beta-NGF), and fibroblast growth factor 19 (FGF-19).

### Causal relationships between immunity and sarcopenia (based on FDR)

**Figure 5:**
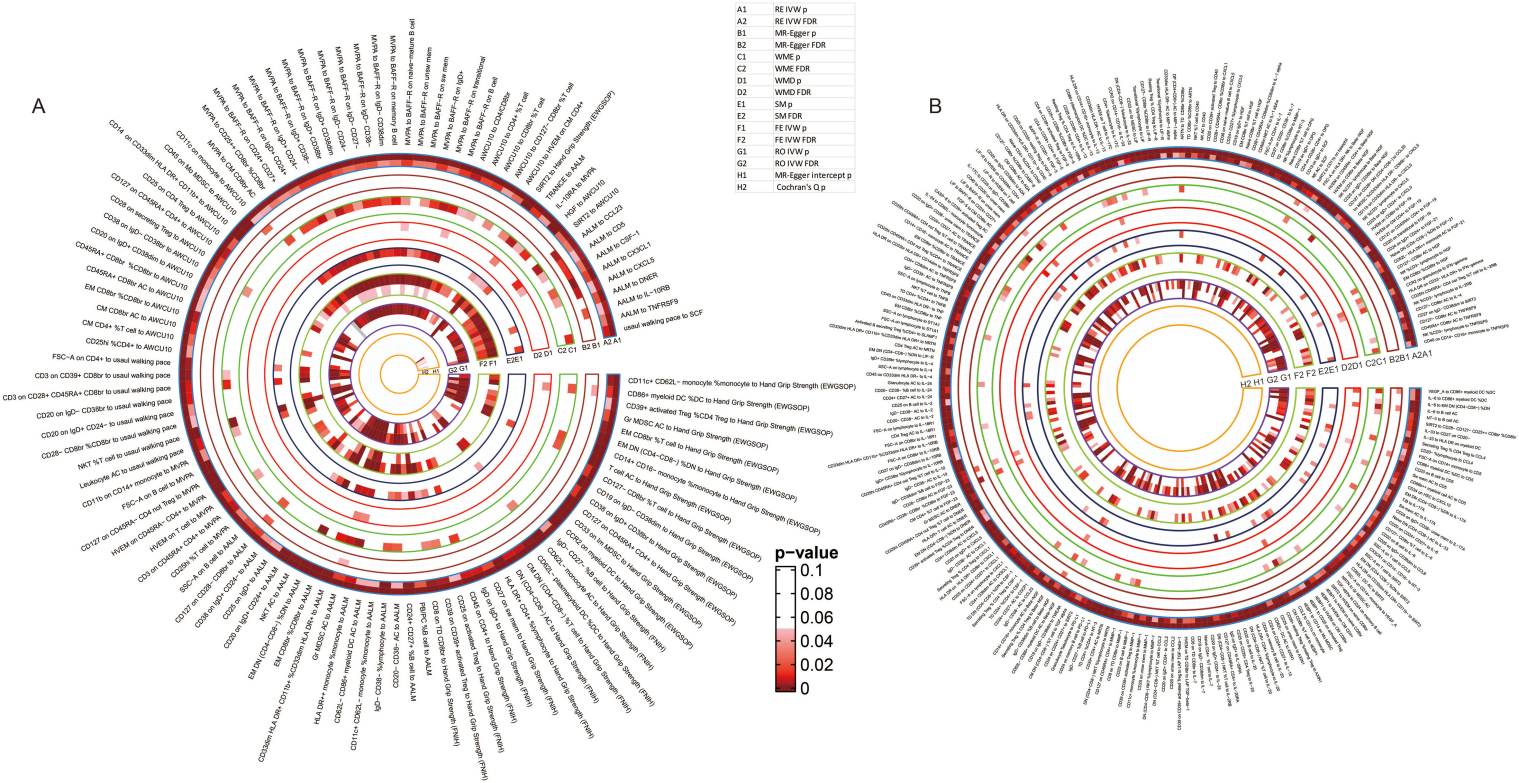
Detailed results of the causal relationships with significant FDR. **(A)** the significant causal relationships between immunity traits and sarcopenia traits; **(B)** the significant causal relationships between immune cell phenotypes and immune proteins.

To avoid the bias caused by multiple tests, we further adjusted the results by using B-H method and mapped the terser landscapes threshold of FDR < 0.05. After the correction, 47, 67, 96, 65, 34, and 54 causal associations with significant FDR between immune cell phenotypes, proteins, and sarcopenia traits were identified in the 6 sarcopenia traits, respectively. The visualization of the results of significant associations, including the sensitivity analyses, was shown in **Figure 5**: the associations with sarcopenia traits were summarized in **Figure 5A**, and the causal relationships between immune cell phenotypes and proteins were visualized in **Figure 5B**.

Specifically, 14 cell phenotypes and SIRT2 were identified to be causally associated with hand grip strength (EWGSOP), and 32 associations between cell phenotypes and proteins were still significant after adjustment of the p values (**Supplementary Table 58**). For hand grip strength (FNIH), 12 cell phenotypes and 55 cell-protein associations were still significant after the correction (**Supplementary Table 59**). The causal association of AALM with 17 cell phenotypes and 9 proteins and 70 causal relationships between cells and proteins were still with significant FDR (**Supplementary Table 60**). Moreover, 26 immune cells and IL-10RA were estimated to have significant associations with MVPA, and 38 causal relationships were still significant after adjusting the p values (**Supplementary Table 61**). For the usual walking pace trait, the relationships between 8 cell phenotypes, Stem cell factor (SCF), and usual walking pace were proven, and 25 causal associations between cells and proteins were still with significant FDR (**Supplementary Table 62**). Lastly, the associations of 19 cells and 2 proteins with AWCU10 and 33 cell-protein causal associations were with FDR < 0.05 (**Supplementary Table 63**).

### The causal network between immunity and sarcopenia (based on FDR)

**Figure 6:**
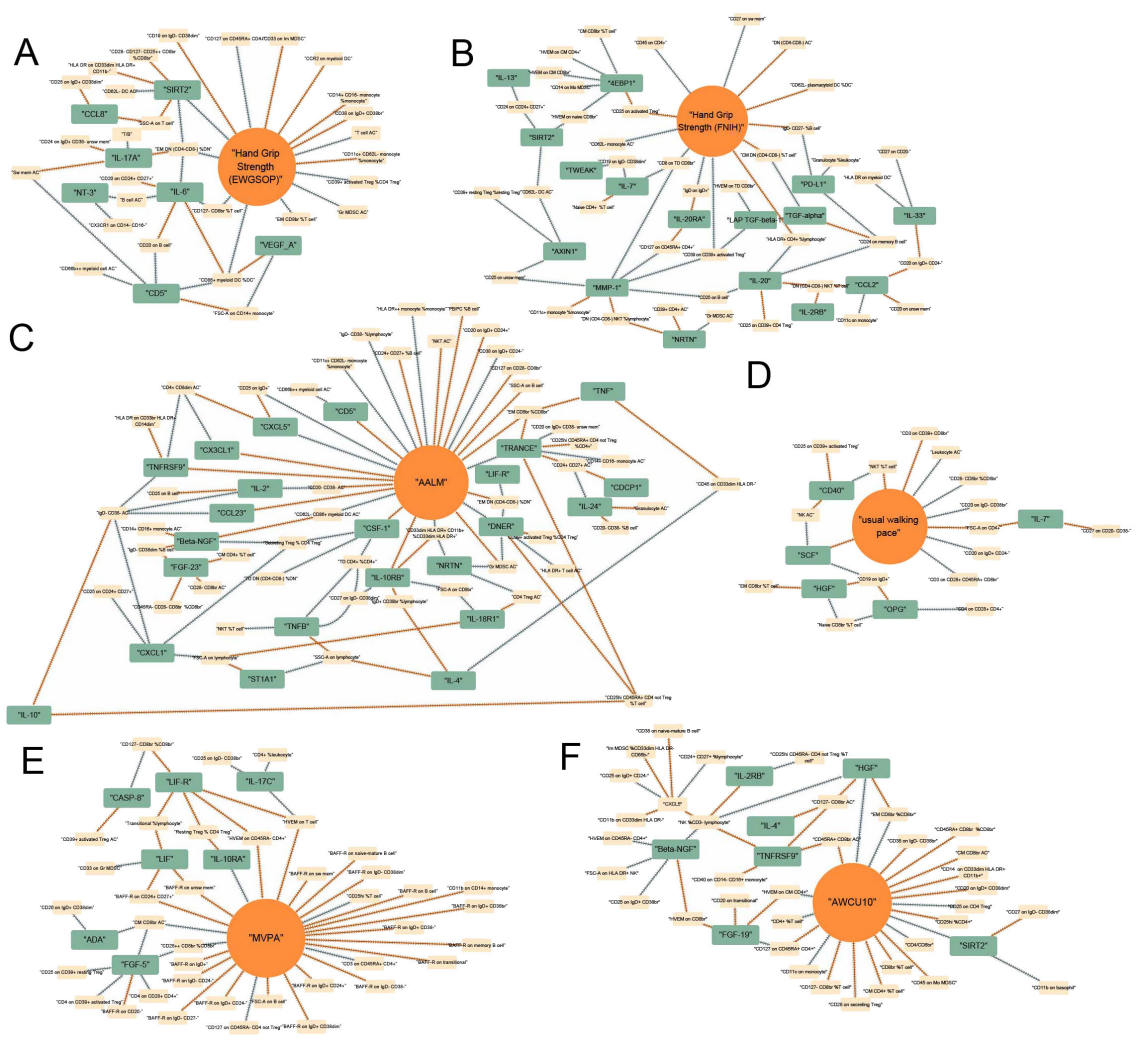
Causal networks between immunity and sarcopenia (based on FDR). **(A)** of hand grip strength (EWGSOP); **(B)** of hand grip strength (FNIH); **(C)** of adjusted appendicular lean mass (AALM); **(D)** of moderate-to-vigorous physical activity (MVPA); **(E)** of usual walking pace; **(F)** of able to walk or cycle unaided for 10 minutes (AWCU10).

Integrating the results of the causal associations with significant FDR, we tried to finally map the landscapes of the causal pathways between immunity and sarcopenia. In these landscapes, all the items, including immune cell phenotypes, immune proteins, and sarcopenia traits, have significant associations with other traits. The causal networks grouped by different sarcopenia traits were visualized in **Figures 6A–F**. The line with an arrow between the two points represents a significant causal relationship, with the arrow pointing from the exposure to the outcome, and the color representing the positive and negative effects of the causal effect, with orange indicating positive regulation and blue indicating negative regulation. By interpreting the networks, we may estimate the pathways between immunity and sarcopenia.

The top traits with the highest rank-sum in the networks are summarized in **Figure 4**. The results of the centrality of traits evaluated by 12 algorithms in networks for 6 sarcopenia traits in the threshold of FDR were summarized in **Supplementary Tables 64**–**69**. The orders of traits ranked by their weights in each algorithm were summarized in **Supplementary Tables 70**–**75**. The ranks and rank sums of traits were summarized in **Supplementary Tables 76**–**81**. In the networks of hand grip strength (EWGSOP), the top traits included relative count of effector memory CD4^-^ CD8^-^ T cells in CD4^-^ CD8^-^ T cells (EM DN (CD4- CD8-) %DN), SIRT2, interleukin-6 (IL-6), and so on. For hand grip strength (FNIH), the top 3 traits were Matrix metalloproteinase-1 (MMP-1), interleukin-20 (IL-20), and MFIs of CD8 on terminally differentiated CD8^bright^ T cells (CD8 on TD CD8br). The top three immune traits in AALM-network were tumor necrosis factor-related activation-induced cytokine (TRANCE), IL-10RB, and Delta and Notch-like epidermal growth factor-related receptor (DNER). In the network of MVPA, MFIs of tumor necrosis factor ligand superfamily member 14 on T cell (HVEM on T cell), absolute count of central memory CD8^bright^ T cells (CM CD8br AC), and relative count of CD25^++^ CD8^bright^ Tregs in CD8^bright^ Tregs (CD25++ CD8br %CD8br) were the top traits. In the causal networks of usual walking pace, the traits with the highest rank sums were SCF, MFIs of CD19 on IgD^+^ B cells (CD19 on IgD+), forward scatter of CD4+ T, B, and NK cells (FSC-A on CD4+), and so on. HGF, relative count of NK cells in CD3^-^ lymphocyte (NK %CD3- lymphocyte), and relative count of effector memory CD8^bright^ T cells in CD8^bright^ T cells (EM CD8br %CD8br) were the traits with highest rank sums in the network of AWCU10.

### Comprehensive causal network between immunity and sarcopenia

**Figure 7:**
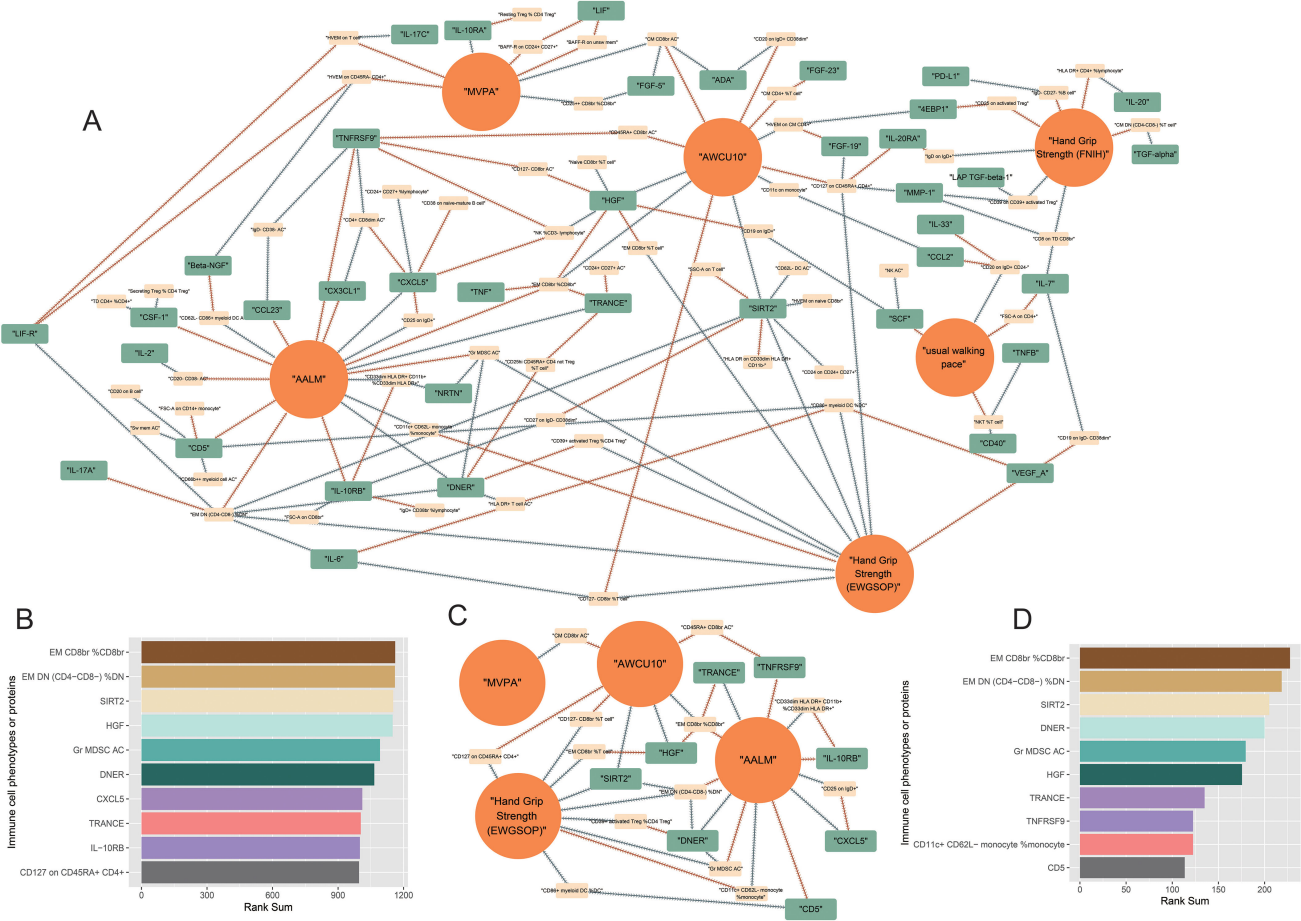
Final comprehensive causal maps. **(A)** comprehensive map between immunity and all sarcopenia traits; **(B)** Top 10 immune traits with the highest rank-sum in the comprehensive map **(A)**; **(C)** final comprehensive map between immunity and all sarcopenia traits (the linkages that were not directly to sarcopenia traits were omitted); **(D)** Top 10 immune traits with the highest rank sums in the final comprehensive map **(C)**.

To obtain a more comprehensive and concise landscape of the causal pathways, we further integrated the networks of all sarcopenia traits and omitted the traits that only causally related to only one sarcopenia trait and that may not contribute to pathways. The comprehensive landscape is summarized in **Figure 7A**. 39 immune proteins and 61 immune cell phenotypes were included in this network. The detailed results of this map were summarized in **Supplementary Tables 82**–**84**. The top 10 traits with the highest rank sums were visualized in **Figure 7B**, including EM CD8br %CD8br, EM DN (CD4- CD8-) %DN, and SIRT2.

To achieve the highest predictive power, we further adjusted the map. The immune cell phenotypes and proteins with less than two causal associations and that were not directly associated with sarcopenia traits after the B-H correction were excluded in the landscapes (**Figure 7C**). The results of the evaluation of the centrality of traits were summarized in **Supplementary Tables 85**–**87** and **Figure 7D**.

In this final map, 13 immune cell phenotypes and 8 immune proteins were included. The cell phenotypes included CM CD8br AC, absolute count of CD45RA^+^ CD8^bright^ T cells (CD45RA+ CD8br AC), relative count of CD127^-^ CD8^bright^ Tregs in T cells (CD127- CD8br %T cell), MFIs of CD127 on CD45RA^+^ CD4^+^ Tregs (CD127 on CD45RA+ CD4+), relative count of effector memory CD8^bright^ T cells in T cells (EM CD8br %T cell), EM CD8br %CD8br, relative count of CD33^dim^ HLA DR^+^ CD11b^+^ myeloid cells in CD33^dim^ HLA DR^+^ myeloid cells (CD33dim HLA DR+ CD11b+ %CD33dim HLA DR+), EM DN (CD4- CD8-) %DN, MFIs of CD25 on IgD^+^ B cells (CD25 on IgD+), relative count of CD39^+^ activated Tregs in CD4 Tregs (CD39+ activated Treg %CD4 Treg), absolute count of granulocytic myeloid-derived suppressor cells (Gr MDSC AC), relative count of CD86^+^ myeloid dendritic cells in dendritic cells (CD86+ myeloid DC %DC), relative count of CD11c^+^ CD62L^-^ monocytes in monocytes (CD11c+ CD62L- monocyte %monocyte). The 8 immune proteins included in the final map were SIRT2, IL-10RB, DNER, CD5, C-X-C motif chemokine 5 (CXCL5), TRANCE, tumor necrosis factor receptor superfamily member 9 (TNFRSF9), HGF.

## Discussion

Inflammaging is associated with increased levels of pro-inflammatory cytokines, such as IL-6 and TNF-alpha, as well as alterations in immune cell phenotypes (19). These inflammatory changes can negatively impact muscle protein synthesis and breakdown, leading to muscle wasting and weakness (20). Understanding the mechanisms underlying inflammaging in sarcopenia is crucial for developing targeted interventions to mitigate its effects (21). Identifying the specific roles of immune cells and proteins in the context of inflammaging can provide valuable insights into the pathophysiology of sarcopenia and inform the development of novel therapeutic strategies.

In this study, by setting different thresholds, we comprehensively established the causal networks between immunity and sarcopenia by using MR. These maps contained multiple dimensions of knowledge in the relationships between immunity and sarcopenia. In the one-dimensional relationships, or the two-variable relationships, the causal associations of single immunity traits, such as immune cell phenotypes and the immune proteins, with sarcopenia traits were the fundamental linkages in the whole maps, which may indicate the direct causal relationships between immunity and sarcopenia. In this dimension, we may directly identify the immune cell phenotypes and proteins that may play the roles of exposures or outcomes in the development of sarcopenia.

However, the understanding of two-variable dimensions was still limited. The systems in the body are intricate and interactional, and various pathways with specific roles are cross-linked to each other. We tried to interpret the relationships between immunity and sarcopenia in a higher dimension. By integrating the relationships between immune cell phenotypes, immune proteins, and sarcopenia traits, we further expand the linkages. In this map with higher dimensions, in addition to the direct immune traits with causal associations of sarcopenia, the deeper traits that may causally relate to the direct immune traits were also identified. Though some cell phenotypes and proteins may not directly associate with sarcopenia, they may also have causal effects on sarcopenia by mediating the direct traits.

The maps always contained a large number of causal associations so we must use a method to identify the key traits. Though under the threshold of p values < 0.05 the causal relationships were all significant, the traits that played more roles in the map and that were included in more relationships were considered as key traits. We used 12 usual algorithms to quantify the centrality of the traits in the causal relationships, and developed a method based on rank sums to finally test the weights of these traits. Therefore, the traits with higher rank sums may have much more roles in the networks between immunity and sarcopenia, and their specific roles need further exploration.

In the final comprehensive map, we only included the traits that directly relate to sarcopenia traits (FDR < 0.05). In this estimate, instead of the test for the centrality of the traits and identifying the significance of the traits in the whole network, we may more concentrate on the credibility and predictive power of the results. The pathways formed by cell phenotypes and proteins that were all directly and causally related to sarcopenia traits in the final map may more credibly reflect the causal networks.

Many interesting pathways were included in the comprehensive maps. EM CD8br %CD8br might be a critical cell phenotype due to its high centrality degree in the map. EM CD8br %CD8br presents the relative count of effector memory CD8^bright^ T cells in CD8^bright^ T cells. In the comprehensive map (**Figure 7A**), the higher relative count of EM CD8 CD8^bright^ T cells in CD8^bright^ T cells was causally related to higher levels of HGF, TNF, TRANCE(RANKL), and higher scores of AALM, and lower ability of AWCU10. The causal effects of EM CD8br %CD8br in our study seemed controversial. Higher EM CD8br %CD8br had directly causal effects on higher AALM, while EM CD8br %CD8br may causally related to higher levels of RANKL, and higher levels of RANKL were directly associated with lower AALM. The risk effects of RANKL on sarcopenia have been proven by previous studies, and RANKL has been identified as a critical osteokine, which proved the stability and credibility of our causal network (22). In this network, we further expand the upstream cell phenotypes of RANKL, including EM CD8br %CD8br and the absolute count of CD24^+^ CD27^+^ B cells (CD24+ CD27+ AC). Back to EM CD8br %CD8br, the phenotype may causally influence the AWCU10 directly or via HGF. Our network suggested that higher EM CD8br %CD8br may causally relate to higher HGF levels and low ability of AWCU10 directly, and the higher HGF also had negative causal effects on AWCU10. The effects of HGF on sarcopenia have been reported by experimental studies (23). In this map, we further identified the potential cell phenotypes with causal associations with HGF, including relative count of naïve CD8^bright^ T cells in T cells (Naive CD8br %T cell), absolute count of CD127^-^ CD8^bright^ Tregs (CD127- CD8br AC), relative count of NK cells in CD3^-^ lymphocytes (NK %CD3- lymphocyte), EM CD8br %CD8br, and EM CD8br %T cell.

Another significant cell phenotype in our maps is EM DN (CD4- CD8-) %DN, which represents the relative count of effector memory CD4^-^ CD8^-^ T cells in CD4^-^ CD8^-^ T cells. In this study, higher EM DN (CD4- CD8-) %DN was proven to be causally associated with higher levels of IL-17A, higher scores of AALM, lower levels of SIRT2, LIF-R, and DNER, and lower risk of poor hand grip strength (EWGSOP), which might be protective causal factors of sarcopenia. The protective causal effects of EM DN (CD4- CD8-) %DN on sarcopenia were also possible via SIRT2, due to the negative causal association of SIRT2 with AWCU10. In the comprehensive map, EM DN (CD4- CD8-) %DN was causally related to IL-6, and higher levels of IL-6 may causally contribute to the low EM DN (CD4- CD8-) %DN, which may inhibit the protective roles of EM DN (CD4- CD8-) %DN. IL-6 has been proven as a significant factor in sarcopenia (24), and the hypothesis from our study adds a new mechanism for IL-6 in the development of sarcopenia.

In addition to EM DN (CD4- CD8-) %DN, SIRT2 was also proven to be causally related to multiple cell phenotypes, including MFIs of CD27 on IgD^-^ CD38^dim^ B cells (CD27 on IgD- CD38dim), side scatters of T cells (SSC-A on T cell), absolute count of CD62L^-^ dendritic cells (CD62L- DC AC), MFIs of tumor necrosis factor ligand superfamily member 14 on naïve CD8^bright^ T cells (HVEM on naïve CD8br), MFIs of CD24 on CD24^+^ CD27^+^ B cells (CD24 on CD24+ CD27+), MFIs of HLA DR on CD33^dim^ HLA DR^+^ CD11b^-^ myeloid cells (HLA DR on CD33dim HLA DR+ CD11b-). However, the roles of SIRT2 in sarcopenia seemed paradoxical: the causal effects of SIRT2 on hand grip strength were protective while on AWCU10 was negative. Previous studies indicated that SIRT2 may regulate the transcriptional expression of myogenic regulatory factors and positively contribute to impaired muscle regeneration and muscle atrophy (25). Most importantly, SIRT2 was proven to be a critical factor in inflammaging due to its role as an acetylation switch of NLPR3 (26). In this study, the potential upstream cell phenotypes were identified and the downstream effects of SIRT2 on sarcopenia were also enriched.

Some limitations should be considered when interpreting the results of our study. Firstly, all participants in our study were of European descent, and the conclusions may be not accurate for populations of other descent. Secondly, we set RE IVW as the main MR model in our study, and some significant results in other models may be ignored, but we have summarized the results of other MR models in the **Supplementary Materials**, which may help to explore the potential causal linkages estimated by other models. Lastly, to satisfy the basic assumptions of MR, we set several steps for IVs selection. Though these steps may improve the power of our analyses, the number of IVs may be limited.

This is the first study that comprehensively mapped the landscape of the causal relationships between immune cell phenotypes, immune proteins, and sarcopenia traits by using MR models. Not limited to the relationships between two variables, this study further linked all the potential causal associations and established the networks, which may reveal the pathways and linkages between immunity and sarcopenia from a connective and general view. Furthermore, discerning the causal links within these networks can facilitate the development of personalized strategies for preserving muscle health and enhancing immune function in aging populations. A comprehensive understanding of how immune processes influence skeletal muscle integrity and vice versa promises to inform precision medicine approaches tailored to individuals at risk of sarcopenia, ultimately leading to improved clinical outcomes and quality of life in elderly individuals.

## Data Availability

The raw data of immune cell traits could be found in the GWAS Catalog with accession numbers from GCST90001391 to GCST90002121. The raw data of immune protein traits could be found in the GWAS Catalog with accession numbers from GCST90274758 to GCST90274848. The accession number for each trait is reported in **Supplementary Tables 1-3**. The raw data of hand grip strength (EWGSOP) and hand grip strength (FNIH) could be found in the GWAS Catalog with accession numbers from GCST90007526 and GCST90007529. The raw data of AALM could be found in the GWAS Catalog with accession numbers from GCST90000025. The raw data of MVPA could be found in the GWAS Catalog with accession numbers from GCST006097. The raw data of usual walking pace could be found in the GWAS Catalog with accession numbers from ukb-b-4711. The raw data of AWCU10 could be found in the GWAS Catalog with accession numbers from ukb-b-14149.
